# Validation of the Patient-Reported Outcomes Measurement Information System (PROMIS^®^) physical function questionnaire in late-onset Pompe disease using PROPEL phase 3 data

**DOI:** 10.1186/s41687-024-00686-z

**Published:** 2024-01-31

**Authors:** Priya S. Kishnani, Simon Shohet, Syed Raza, Noemi Hummel, Jeffrey P. Castelli, Sheela Sitaraman Das, Heng Jiang, Agnieszka Kopiec, Ian Keyzor, Andreas Hahn

**Affiliations:** 1 Duke University, 905 Lasalle Street, GSRB1, Room 4010, Durham, NC 27710 USA; 2 Amicus Therapeutics UK LTD, One Globeside, Fieldhouse Ln, Marlow, SL7 1HZ UK; 3 Argenx BV Belgium, Industriepark Zwijnaarde 7, Gent, 9052 Belgium; 4 Certara GmbH Germany, Chesterplatz 1, 79539 Lörrach, Germany; 5 Amicus Therapeutics, 47 Hulfish St, Princeton, NJ 08542 USA; 6 Certara France, 69–71 rue de Miromesnil, Paris, 75008 France; 7 Certara Poland, Kuklinskiego 17, 30-720, Krakow, Poland; 8 Justus-Liebig-University, Feulgenstr. 10-12, 35392 Giessen, Gießen, Germany

**Keywords:** Patient-reported outcomes, Patient-Reported Outcome Measurement Information System (PROMIS), Late-onset Pompe disease, Physical function, Quality of life, PROPEL, Validation

## Abstract

**Background:**

The construct validity and interpretation of the Patient-Reported Outcome Measurement Information System (PROMIS^®^) Physical Function short form 20a (PF20a) questionnaire were evaluated for patients with late-onset Pompe disease (LOPD), a rare, autosomal recessive, progressive neuromuscular disorder treatable by enzyme replacement therapy (ERT).

**Methods:**

In the phase 3 PROPEL study, adults with LOPD underwent testing of physical functioning and had PRO measurements at baseline and at weeks 12, 26, 38, and 52 while receiving experimental or standard-of-care ERT. All patients were pooled for analyses, without comparisons between treatment groups. Associations and correlations between PROMIS PF20a scores and the 6-minute walk distance (6MWD), % predicted forced vital capacity (FVC), manual muscle test (MMT) of the lower extremities, Gait, Stairs, Gowers’ maneuver, Chair (GSGC) score, and Rasch-built Pompe-specific Activity (R-PAct) scale were evaluated by calculating regression coefficients in linear regression models and Pearson correlation coefficients (R); patients’ age, sex, race, ERT prior to study, body mass index, and study treatment were included as covariables. The minimal clinically important difference (MCID) of PROMIS PF20a was determined using distribution- and anchor-based methods.

**Results:**

123 patients received at least 1 dose of ERT. In multivariable analyses, PROMIS PF20a scores had strong correlations with R-PAct scores (*R* = 0.83 at baseline and *R* = 0.67 when evaluating changes between baseline and 52 weeks) and moderate correlations with the 6MWD (*R* = 0.57 at baseline and *R* = 0.48 when evaluating changes between baseline and 52 weeks). Moderate correlations were also observed between PROMIS PF20a and MMT (*R* = 0.54), GSGC (*R*=-0.51), and FVC (*R* = 0.48) at baseline. In multivariable linear regression models, associations were significant between PROMIS PF20a and 6MWD (*P* = 0.0006), MMT (*P* = 0.0034), GSGC (*P* = 0.0278), and R-PAct (*P* < 0.0001) at baseline, between PROMIS PF20a and 6MWD (*P* < 0.0001), FVC (*P* = 0.0490), and R-PAct (*P* < 0.0001) when combining all measurements, and between PF20a and 6MWD (*P* = 0.0016) and R-PAct (*P* = 0.0001) when evaluating changes in scores between baseline and 52 weeks. The anchor-based and distribution-based MCID for a clinically important improvement for PROMIS PF20a were 2.4 and 4.2, respectively.

**Conclusions:**

PROMIS PF20a has validity as an instrument both to measure and to longitudinally follow physical function in patients with LOPD.

**Trial registration:**

ClinicalTrials.gov, NCT03729362. Registered 2 November 2018, https://www.clinicaltrials.gov/search?term=NCT03729362.

**Supplementary Information:**

The online version contains supplementary material available at 10.1186/s41687-024-00686-z.

## Abstract (de)

Late-onset Pompe disease (LOPD) is a rare, hereditary disease. Patients with LOPD have decreased production of an enzyme, which leads to symptoms that gradually get worse, including muscle weakness and trouble breathing. Enzyme replacement therapy may slow down the disease progression. In recent years, enzyme replacement therapies have been improved. To measure the benefit of such new therapies, patients with LOPD are asked to fill in surveys about their symptoms before and during treatment, but there is no standard survey to use. In this study, we used a survey called the Patient-Reported Outcome Measurement Information System (PROMIS^®^) Physical Function short form 20a (PF20a) questionnaire. This questionnaire is used for various diseases and tests someone’s ability to perform daily physical activities such as getting dressed. We compared the results of this survey to other tests which evaluate a variety of functions, such as how far a patient can walk in 6 minutes, leg muscle strength, and a patient’s lung capacity. In general, we found that the score provided by the PROMIS PF20a questionnaire had moderate to strong agreement with other test scores. Furthermore, we looked at the minimum difference in PROMIS PF20a scores that a patient found a relevant difference (i.e., a relevant improvement or worsening of the disease). Patients found a difference between 2.4 and 4.2 points in the score relevant. The results from this study show that PROMIS PF20a may be used to measure symptoms and follow symptoms over time in patients with LOPD.

## Background

Pompe disease is a rare, autosomal recessive, metabolic disorder [1]. Due to pathogenic variants in the *GAA* gene, patients have an acid α-glucosidase (GAA) deficiency, leading to accumulation of glycogen in lysosomes. In late-onset Pompe disease (LOPD), patients have some residual enzyme activity (ranging from ~ 1% to 2% up to 40%), resulting in an onset of symptoms ranging from early childhood to late adulthood [2, 3]. Symptoms of LOPD are predominantly related to skeletal muscle and diaphragmatic dysfunction, causing mobility and respiratory difficulties [1]. Treatment consists of enzyme replacement therapy (ERT) to slow disease progression, supplemented by symptomatic treatment and supportive care.

Recently, advances have been made for LOPD treatment with the development of second-generation ERTs using recombinant human GAA (rhGAA), such as avalglucosidase alfa [4, 5] and cipaglucosidase alfa in combination with the small molecule stabilizer miglustat [6]. Avalglucosidase alfa received approval by the United States Food and Drug Administration in 2021 [7] and cipaglucosidase alfa with miglustat received approval by the European Medicines Agency in 2023 [8]. These rhGAAs enhance mannose-6-phosphate receptor-mediated uptake of GAA, resulting in increased clearance of the accumulated glycogen in lysosomes. While not a cure, these agents aim to improve the mobility and respiratory capacity of patients and therefore, to improve the well-being and quality of life of patients with LOPD.

To measure clinically meaningful benefit of rhGAA, patient-reported outcomes (PROs) are used [9, 10]. Examples of PRO measurements previously used in LOPD include the Rotterdam handicap scale, the Medical Outcomes Study 36-Item Short-Form Health Survey (SF-36), the Rasch-built Pompe-specific Activity (R-PAct) scale, the Pompe Disease Symptom Scale (PDSS), and the Pompe Disease Impact Scale (PDIS), the latter 3 having been developed recently to specifically evaluate disease progression and treatment in LOPD [5, 11–14].

As PROs rely on subjective interpretation from patients, PRO measurements need to be validated to ensure they adequately measure relevant and important aspects of the disease, and that limitations of the instrument are known. The extensive recommendations from COnsensus-based Standards for the selection of health Measurement INstruments (COSMIN), driven by an international expert panel, define terminology of measurement properties of PROs and provide guidance evaluating the methodological quality of studies on these measurement properties, recommending assessment of reliability, validity, and responsiveness of PRO measurements [15–17]. The International Society for Quality-of-Life Research (ISOQOL) developed minimum standards for PRO measures combining existing literature, including guidance from COSMIN and health authorities [18]. Using recommendations from ISOQOL, COSMIN, and other relevant literature, Francis et al. have subsequently developed a checklist to operationalize PRO measurements, which includes 18 scoring criteria to evaluate the conceptual model, content validity, reliability, construct validity, scoring and interpretation, respondent burden and presentation of the PRO measurement [19].

Validation of PRO measurements is particularly important in LOPD, as no gold standard of PRO measurement exists to date. In previous studies, some validation checks have been conducted to evaluate the use of R-PAct, PDIS, and PDSS in patients with LOPD [13, 14, 20]. In addition, Harfouche et al. evaluated the use of the PRO Measurement Information System (PROMIS^®^) in 30 patients with Pompe disease, concluding that selected PROMIS questionnaires are meaningful and address important concepts to patients with Pompe disease, including motor function and symptoms of functional disability [21]. However, this study had some limitations, including the low number of patients, open-label design with patients knowing what treatment they were receiving, and that PROMIS was measured at a single time point only. Subsequently, the PROMIS Physical Function short form 20a questionnaire (PROMIS PF20a) was included as a PRO instrument in the PROPEL phase 3 study, its patient-level data providing a key source of cross-sectional and longitudinal data to analyze the questionnaire in LOPD [6]. The PROMIS PF20a measures current self-reported capability of physical activities, including functioning of upper and lower extremities and central regions, and instrumental activities of daily living. It can be used in the adult general population and adults with chronic health conditions, in both clinical trials or clinical practice settings. The aim of this report is to show construct validity of PROMIS PF20a by comparing the scoring of this questionnaire in PROPEL to various tests for physical functioning as well as the R-PAct scale, both cross-sectionally and longitudinally, to further validate its use in LOPD. A further aim was to improve interpretation of the PROMIS PF20a score in patients with LOPD by determining the minimal clinically important difference (MCID) of PROMIS PF20a scores.

## Methods

### Patient selection

To evaluate the PROMIS PF20a questionnaire in LOPD, data from the PROPEL study were used [6]. PROPEL (NCT03729362) is a global, randomized, double-blind, parallel-group, phase 3 clinical trial that evaluated the efficacy and safety of cipaglucosidase alfa plus miglustat (*n* = 85) compared to alglucosidase alfa plus placebo (*n* = 38) in adult patients (age ≥ 18 years, body weight ≥ 40 kg) with confirmed LOPD. Patients were either ERT-naïve or had been treated with alglucosidase alfa for ≥ 2 years (20 mg/kg once every 2 weeks; ERT-experienced). For the analyses in this study, all patients were pooled into 1 cohort without differentiating between treatments, to make use of all available data. A comparison of PROMIS PF20a scores between treatment arms has been conducted as a key secondary endpoint of PROPEL and is described by Schoser et al. [6]. Additional details of the study protocol have been published previously [6].

### Data collection

Patients’ baseline characteristics collected in PROPEL and used in the current study include age, sex, race, body mass index (BMI) and previous ERT status (naïve or experienced). Various outcome measurements were evaluated at baseline, weeks 12, 26, 38, and 52. In this study, we included the following outcomes that were measured in PROPEL:

- PROMIS PF20a [6, 22, 23]: patients answer 20 questions on physical function, which the patient can score from unable to do (1) to being able to do without any difficulties or limitations (5). Hence, the score ranges between 20 and 100, with a higher score indicating better physical functioning.
- 6-minute walk distance (6MWD) [6, 24]: the distance (in meters) a patient can quickly walk within 6 min on a flat surface with walking shoes; walking aids (e.g., a cane, walker, or rollator) were permitted and were used consistently throughout the study, when required.
- % predicted forced vital capacity (FVC) [6, 25]: the volume of a maximal forced expiratory effort (FVC) after maximal inspiration, while sitting, compared to the FVC for healthy adults in the National Health and Nutrition Examination Survey (NHANES) III.
- Manual Muscle Test (MMT) of the lower extremities [6, 26]: skeletal muscle strength in the hips and knees is scored using the Medical Research Council scale (0 to 5 points, with a score of 5 indicating normal function and a score of 0 indicating no muscle movement). The final score is the sum of the score for hip flexion and abduction, and knee flexion and extension in both extremities, and therefore, ranges from 0 to 40, with a higher score indicating better muscle function.
- Gait, Stairs, Gowers’ maneuver, Chair (GSGC) score [6, 27]: the patient walks 10 m (gait), climbs 4 stairs, performs the Gowers’ maneuver (begin lying down on the floor, then rise from the floor to a standing position), and stands up from a chair. Each item is scored from 1 (normal function) to 7 (gait: confined to wheelchair; stairs: unable to climb stairs; Gowers’ maneuver: unable to rise) or 6 (chair: unable to get up from chair) and therefore, ranges from 4 to 27, with a lower score indicating better physical functioning.
- R-PAct [13]: patients answer a questionnaire of 18 questions to quantify the effect of Pompe disease on their daily activities and social participation. Three answers are possible: 0 = no; 1 = yes, but with difficulty; 2 = yes, without difficulty. Hence, the score ranges from 0 to 36, with a higher score indicating fewer limitations in activities and social participation.
- Subject’s global impression of change (SGIC) in overall physical well-being [6, 28–30]: the patients answer this question using a 7-point rating scale, with answers ‘1 = very much worse’, ‘2 = worse’, ‘3 = somewhat worse’, ‘4 = no change’, ‘5 = somewhat improved’, ‘6 = improved’, and ‘7 = very much improved’.

### Evaluation of the PROMIS PF20a questionnaire

To analyze construct validity of the PROMIS PF20a questionnaire, correlation and associations were evaluated between PROMIS PF20a and 6MWD, % predicted FVC, MMT of the lower extremities, GSGC, and R-PAct scores, at baseline, combining all measurements (baseline, weeks 12, 26, 38, and 52), and for changes of scores between baseline and 52 weeks.

Additionally, interpretation of changes in PRO measurements was assessed by determining what represented an MCID in PROMIS PF20a. The MCID has previously been defined as “the smallest difference in score in the domain of interest which patients perceive as beneficial and which would mandate, in the absence of troublesome side effects and excessive cost, a change in the patient’s management” [31, 32]. A distribution-based and anchor-based MCID was calculated. In the anchor-based approach, an external measure is used as an anchor, which has established cut-offs to define clinically meaningful improvement and correlates with the measure for which a MCID will be derived. The SGIC in overall physical well-being was selected as an anchor, as it directly asked patients participating in PROPEL whether they had observed a meaningful benefit (or worsening) in the first year after initiating treatment.

### Statistical analyses

For descriptive statistics, categorical variables were summarized by frequency (number of patients) and percentage. Continuous variables were summarized using mean, standard deviation (SD), median, and range.

To evaluate associations between scoring of the PROMIS PF20a and functional measures (6MWD, % predicted FVC, MMT of the lower extremities, and GSGC) or R-PAct, multivariable (adjusted) linear regression models were applied with PROMIS PF20a as the dependent and the respective other measure as independent variable. The models adjusted for age (continuous), sex (categorical: male, female), race (categorical: White, Asian, other), previous ERT status (categorical: naïve, experienced), BMI (continuous), and study treatment (categorical: cipaglucosidase alfa plus miglustat, alglucosidase alfa plus placebo). Regression coefficients (B) including their 95% confidence intervals and p-values were calculated. Additionally, mixed-effects linear regression models, which adjusted for repeated measures at baseline, weeks 12, 26, 38, and 52 within individuals were applied. To evaluate correlations, scatterplots including regression lines were drawn, and the Pearson correlation coefficient (R) was calculated, both for baseline scores and changes in scores after one year of treatment (difference between scores in week 0 and week 52). A Pearson correlation coefficient ≤|0.19| (absolute values, i.e., R between -0.19 and 0.19) was considered very weak correlation, a coefficient ≥|0.20| to ≤|0.39| (i.e., -0.39 to -0.20 or 0.20 to 0.39) a weak correlation, a coefficient ≥|0.40| to ≤|0.59| a moderate correlation, a coefficient ≥|0.60| to ≤|0.79| a strong correlation, and a coefficient ≥|0.80| a very strong correlation [33]. In the main analyses, Pearson correlation coefficients were adjusted for abovementioned covariables; sensitivity analyses excluded these variables from the models (unadjusted models).

The distribution-based MCID was calculated by taking 1/3 of the SD of the PROMIS PF20a scores at baseline of all PROPEL patients [34–36]. The anchor-based MCID for improvement and deterioration were defined as the mean change from baseline in PROMIS PF20a scores in patients who had reported that their overall physical well-being had somewhat improved (SGIC score = 5) or somewhat worsened (SGIC score = 3) at week 52, respectively [31]. As a sensitivity analysis, the MCID was defined as the mean change in PROMIS PF20a scores in patients who had reported that their overall physical well-being remained stable (SGIC score = 4).

All statistical analyses were conducted using SAS (version 9.4 TS1M4).

## Results

In total, 125 patients were randomized in PROPEL, and 123 patients were dosed with cipaglucosidase alfa plus miglustat (*n* = 85) or alglucosidase alfa plus placebo (*n* = 38). Mean age of these 123 patients was 47 years (SD 13 years), patients had a mean BMI of 25 kg/m^2^ (SD 6 kg/m^2^), 55% of the patients were female, most patients (85%) were White and 77% had previously received ERT (Supplementary Table 1). In the outcomes analyses, 1 patient was excluded as the patient deliberately underperformed at baseline to gain entry into the study [6]. Results from outcomes measuring physical function and PROs in the PROPEL study are summarized in Table 1. At baseline, having pooled patients from both treatment groups into 1 cohort, the mean PROMIS PF20a score was 67 (SD 12, *n* = 121), patients walked a mean distance of 356 m (SD 114 m, *n* = 122) in the 6MWD test, had a % predicted FVC of 70 (SD 20, *n* = 122), and scored on average 28 (SD 6, *n* = 118), 15 (SD 5, *n* = 106), and 20 (SD 6, *n* = 102) on the MMT of lower extremities, GSGC, and R-PAct scores, respectively. After 52 weeks, the mean PROMIS PF20a, MMT of lower extremities, GSGC, and R-PAct scores changed to 69 (SD 14, *n* = 121), 29 (SD 6, *n* = 114), 14 (SD 5, *n* = 102), and 21 (SD 7, *n* = 102), respectively; patients walked a mean distance of 372 m (SD 127, *n* = 122) in the 6MWD test, and had a % predicted FVC of 69 (SD 20, *n* = 121).

**Table 1:** Summary of outcome measurements collected in PROPEL

Outcome	Baseline	Week 12	Week 26	Week 38	Week 52	Δ baseline and week 52
PROMIS Physical Function short form 20a score						
N	121	117	121	121	121	121
mean, SD	67 (12)	69 (14)	69 (14)	69 (14)	69 (14)	1 (9)
median, range	67 (37–97)	70 (37–98)	69 (32–99)	70 (36–99)	68 (36–99)	0 (-30–30)
6-minute walk distance in meters						
N	122	122	122	122	122	122
mean, SD	356 (114)	367 (119)	370 (123)	370 (122)	372 (127)	17 (42)
median, range	363 (79–623)	374 (101–619)	378 (40–678)	376 (77–634)	378 (67–649)	11 (-60–174)
FVC % predicted						
N	122	121	120	120	121	121
mean, SD	70 (20)	70 (21)	69 (20)	69 (20)	69 (20)	-2 (6)
median, range	70 (31–133)	69 (32–155)	69 (32–148)	68 (31–138)	69 (28–137)	-1.5 (-20–14)
MMT of lower extremities score						
N	118	113	112	114	114	114
mean, SD	28 (6)	29 (6)	29 (6)	29 (6)	29 (6)	1 (3)
median, range	28 (14–40)	28 (13–40)	29 (13–40)	29 (12–40)	30 (10–40)	1 (-10–18)
GSGC score						
N	106	100	100	100	102	102
mean, SD	15 (5)	14 (5)	14 (5)	14 (5)	14 (5)	0 (2)
median, range	16 (4–24)	16 (4–23)	16 (4–24)	15 (4–24)	16 (4–24)	0 (-8–5)
R-PAct score						
N	102	102	102	102	102	102
mean, SD	20 (6)	21 (7)	21 (6)	20 (7)	21 (7)	0 (3)
median, range	20 (10–36)	21 (8–36)	19 (8–36)	19 (9–36)	19 (8–36)	0 (-9–11)

Δ: difference; FVC: forced vital capacity; GSGC: Gait, Stairs, Gowers’ maneuver, Chair; MMT: manual muscle test; N: number of patients; R-PAct: Rasch-built Pompe-specific Activity scale; SD: standard deviation

In univariable linear regression models, PROMIS PF20a scores were significantly associated with all other outcome measures (Table 2), both at baseline (*P* ≤ 0.0004) and when combining all measurements (*P* ≤ 0.0023). As expected, associations were negative between PROMIS PF20a and GSGC scores, but positive for all other outcomes, as a higher GSGC score indicates worse physical functioning while in all other outcomes a higher score indicates better functioning. PROMIS PF20a scores were also significantly associated with 6MWD (B = 0.09, 95% CI 0.05 to 0.12; per 1-meter increment in the 6MWD test) and R-PAct (B = 1.07, 95% CI 0.65 to 1.50; per 1-point increment) when evaluating changes in scores between baseline and 52 weeks.

**Table 2:** Comparison of PROMIS physical function to other tests evaluating (physical) functioning in LOPD

		PROMIS Physical Function short form 20a			
		Univariable		Multivariable^3^	
		Regression coefficient B (95% confidence interval)	P-value	Regression coefficient B (95% confidence interval)	P-value
6MWD (per 1-meter increment)	0 weeks (baseline)^1^	**0.06** **(0.04, 0.07)**	**< 0.0001**	**0.04** **(0.02, 0.07)**	**0.0006**
	Change from 0 to 52 weeks^1^	**0.09** **(0.05, 0.12)**	**< 0.0001**	**0.08** **(0.03, 0.12)**	**0.0016**
	All measurements^2^	**0.06** **(0.05, 0.07)**	**< 0.0001**	**0.05** **(0.03, 0.06)**	**< 0.0001**
FVC (per 1% increment in predicted value)	0 weeks (baseline)^1^	**0.20** **(0.09, 0.30)**	**0.0004**	0.10 (-0.04, 0.24)	0.1719
	Change from 0 to 52 weeks^1^	0.05 **(**-0.21, 0.31**)**	0.7035	0.06 (-0.30, 0.42)	0.7522
	All measurements^2^	**0.14** **(0.07, 0.21)**	**0.0001**	**0.08** **(0.00, 0.17)**	**0.0490**
MMT of lower extremities (per 1-point increment)	0 weeks (baseline)^1^	**0.96** **(0.61, 1.30)**	**< 0.0001**	**0.67** **(0.23, 1.11)**	**0.0034**
	Change from 0 to 52 weeks^1^	0.15 (-0.33, 0.63)	0.5349	0.08 (-0.45 0.61)	0.7626
	All measurements^2^	**0.26** **(0.10, 0.43)**	**0.0023**	0.11 (-0.07 0.29)	0.2401
GSGC test score (per 1-point increment)	0 weeks (baseline)^1^	**-1.31** **(-1.72, -0.90)**	**< 0.0001**	**-0.78** **(-1.47, -0.09)**	**0.0278**
	Change from 0 to 52 weeks^1^	-0.28 (-1.01, 0.45)	0.4482	-0.30 (-1.30, 0.70)	0.5527
	All measurements^2^	**-0.48** **(-0.74, -0.21)**	**0.0005**	-0.19 (-0.52 0.14)	0.2489
R-PAct (per 1-point increment)	0 weeks (baseline)^1^	**1.81** **(1.57, 2.04)**	**< 0.0001**	**1.72** **(1.36, 2.08)**	**< 0.0001**
	Change from 0 to 52 weeks^1^	**1.07** **(0.65, 1.50)**	**< 0.0001**	**1.03** **(0.52, 1.53)**	**0.0001**
	All measurements^2^	**1.19** **(1.05, 1.34)**	**< 0.0001**	**1.23** **(1.04, 1.41)**	**< 0.0001**

Bold values are those that show statistical significance

6MWD: 6-minute walk distance; FVC: forced vital capacity; GSGC: gait, stairs, Gowers’ maneuver, chair; LOPD: late-onset Pompe disease; MMT: manual muscle test; R-PAct: Rasch-built Pompe-specific Activity scale

^1^Regression coefficients were estimated using uni- and multivariable linear regression models (for unadjusted and adjusted model, respectively)

^2^Regression coefficients were estimated using mixed-effects linear regression models, adjusting for repeated measures within individuals

^3^Regression variables included were treatment (cipaglucosidase alfa plus miglustat or alglucosidase alfa plus placebo), age (continuous), sex (male or female), race (White, Asian, other), enzyme-replacement therapy prior to study (naïve or experienced), and body mass index (continuous) at baseline

Adjusting for age, sex, race, previous ERT status, BMI, and study treatment in multivariable analyses, directionality of associations remained the same. In these multivariable linear regression analyses, baseline PROMIS PF20a scores were significantly associated to baseline 6MWD (B = 0.04, 95% CI 0.02 to 0.07; per 1-meter increment in the 6MWD test), MMT scores of the lower extremities (B = 0.67, 95% CI 0.23 to 1.11; per 1-point increment), GSGC scores (B=-0.78, 95% CI -1.47 to -0.09; per 1-point increment), and R-PAct scores (B = 1.72, 95% CI 1.36 to 2.08; per 1-point increment), but not to the baseline % predicted FVC (B = 0.10, 95% CI -0.04 to 0.24; per 1% increment in predicted FVC). Changes in PROMIS PF20a scores between baseline and 52 weeks were significantly associated with changes in the 6MWD (B = 0.08, 95% CI 0.03 to 0.12) and R-PAct scores (B = 1.03, 95% CI 0.52 to 1.53). Combining measurements at all timepoints in a multivariable, mixed-effects, linear regression model, associations were significant between PROMIS PF20a scores and the 6MWD (B = 0.05, 95% CI 0.03 to 0.06), R-PAct scores (B = 1.23, 95% CI 1.04 to 1.41), and % predicted FVC (B = 0.08, 95% CI 0.00 to 0.17, *P* = 0.049). In summary, these results indicated that PROMIS PF20a scores were associated with other physical function measures, particularly with R-PAct scores and the 6MWD.

**Fig. 1:**
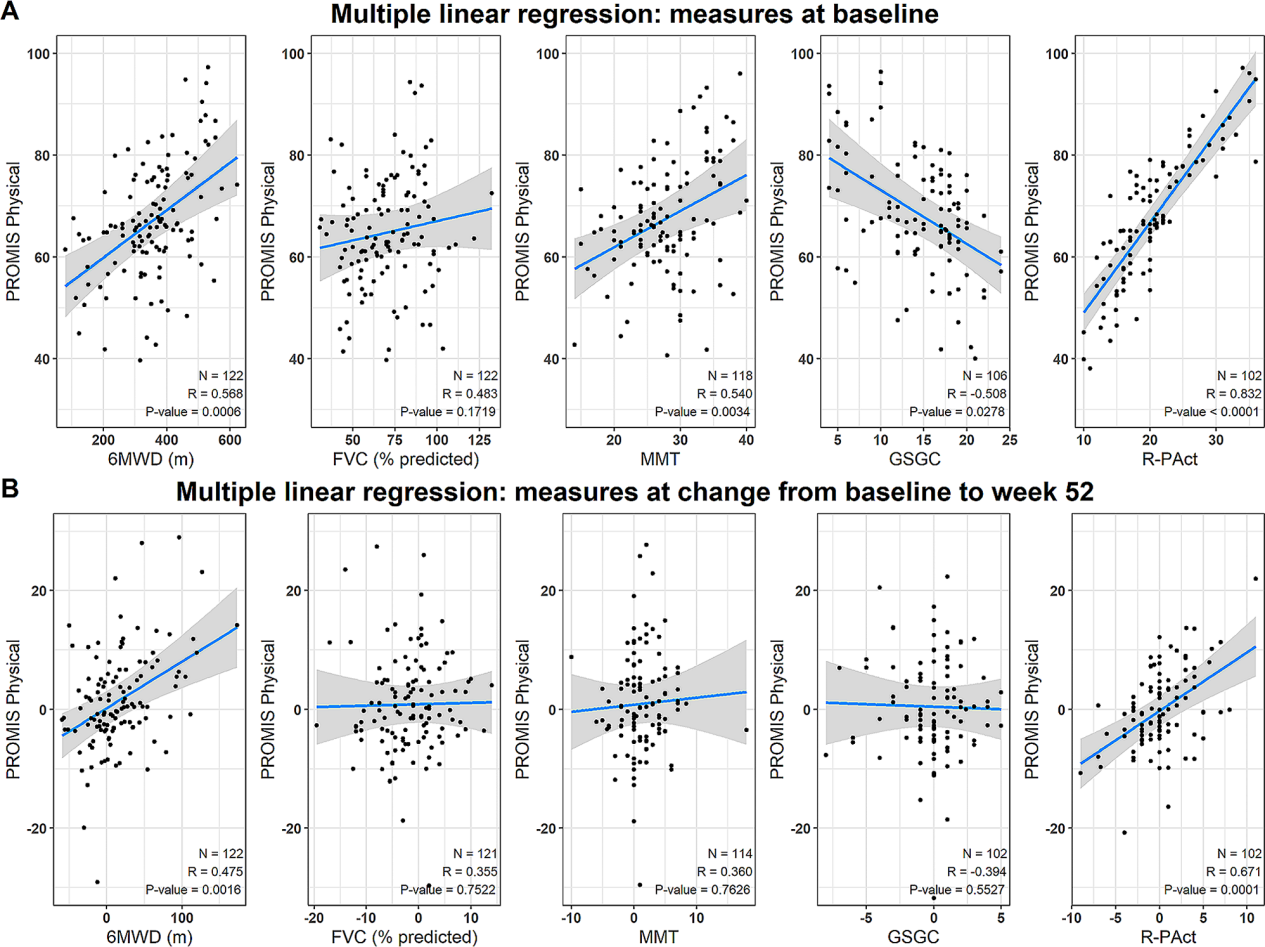
Correlation between PROMIS Physical Function short form 20a scores and other outcome measures, adjusted model. P-values are determined by multivariable linear regression. 6MWD: 6-minute walk distance; FVC: forced vital capacity; GSGC: gait, stairs, Gowers’ maneuver, chair; MMT: manual muscle test; N: number of patients; R: Pearson’s correlation coefficient; R-PAct: Rasch-built Pompe-specific Activity scale

These results were further confirmed by evaluating correlations between PROMIS PF20a and other outcome measures using Pearson’s correlation coefficients (Fig. 1 and Supplementary Fig. 1). In multivariable analyses, a very strong correlation was observed between PROMIS PF20a and R-PAct scores (*R* = 0.832), and moderate correlations were observed between PROMIS PF20a and 6MWD (*R* = 0.568), MMT of the lower extremities (*R* = 0.540), GSGC (*R*=-0.508), and % predicted FVC (*R* = 0.483) at baseline (Fig. 1A). When evaluating changes between baseline and 52 weeks, the correlation between PROMIS PF20a and R-PAct remained strong (*R* = 0.671) and the correlation between PROMIS PF20a and 6MWD remained moderate (*R* = 0.475), whereas the correlations between PROMIS PF20a and GSGC (*R*=-0.394), MMT of the lower extremities (*R* = 0.360), and % predicted FVC (*R* = 0.355) were moderate (Fig. 1B).

In the final analyses, the MCID was determined. The SD of PROMIS PF20a scores at baseline was 12.5, thus, the distribution-based MCID was established at 4.2. Using this cutoff, 28% (*N* = 34) of patients in PROPEL reported an improvement in PROMIS PF20a score after 1 year at or above the MCID (≥ 4.2), and 20% (*N* = 24) were considered to have a clinically important worsening of symptoms (i.e., their PROMIS PF20a scores after 1 year was equal to or below -4.2).

In the anchor-based approach, changes in PROMIS PF20a scores between baseline and week 52 were compared to the SGIC overall physical well-being as reported in week 52. A weak but significant correlation was observed between the two outcome measurements (*R* = 0.2836, *P* = 0.0020) in an unadjusted analysis, and therefore, the SGIC overall physical well-being was considered suitable as an anchor to determine the MCID for the PROMIS PF20a scores.

Patients reporting that their overall physical well-being remained stable (*N* = 45) or improved (*N* = 49), had on average a positive change in PROMIS PF20a scores, ranging from 2.2 (SD 10.0) for those reporting stable overall physical well-being to 8.5 (SD 11.9) for those reporting a much improved overall physical well-being (Table 3). The anchor-based MCID for improvement was established at 2.4, which corresponded to the mean change in PROMIS PF20a score for those patients reporting an improvement (score = 5) in the SGIC overall physical well-being (mean 2.4, SD 6.7). Using this cut-off, 38% (*N* = 46) of patients in PROPEL had a change in PROMIS PF20a score after 1 year compared to baseline equal to or larger than the MCID for improvement (≥ 2.4). The anchor-based MCID for deterioration was established at -3.4, which corresponded to the mean change in PROMIS PF20a score for those patients reporting a worsening (score = 3) in the SGIC overall physical well-being (mean -3.4, SD 7.5). Using this cut-off, 24% of LOPD patients in PROPEL had a clinically important worsening based on their change in PROMIS PF20a score at 1 year compared to baseline.

**Table 3:** Determination of the minimal clinically important difference in PROMIS PF20a scores

Subject’s global impression of change in overall well-being between 0 and 52 weeks	Number of patients (%)	Change in PROMIS PF20a from 0 to 52 weeks, mean (SD)
1: very much worse	0 (0.0)	N/A
2: much worse	4 (3.4)	-2.8 (7.9)
3: somewhat worse	19 (16.2)	-3.4 (7.5)
4: no change	45 (38.5)	2.2 (10.0)
5: somewhat improved	36 (30.8)	2.4 (6.7)
6: much improved	8 (6.8)	4.4 (5.1)
7: very much improved	5 (4.3)	8.5 (11.9)

PROMIS PF20a: Patient-Reported Outcome Measurement Information System Physical Function short form 20a; SD: standard deviation

In the sensitivity analysis, the MCID was established at 2.2, which corresponded to the mean change in PROMIS PF20a score for those patients reporting a stabilization in the SGIC overall physical well-being.

## Discussion

This study evaluated the construct validity and interpretation of the PROMIS PF20a questionnaire in 122 patients with LOPD, using data from the PROPEL phase 3 study [6]. PROMIS PF20a scores generally correlated well and showed statistically significant associations with the functional measures 6MWD, % predicted FVC, MMT, and GSGC, and with R-PAct, in cross-sectional assessments and to a lesser extent in longitudinal assessments. The functional measures evaluated in this study are relevant clinical measures in Pompe disease and are typically included in clinical trials to assess efficacy of treatments in LOPD. Furthermore, outcomes such as the 6MWD are considered relevant in Pompe disease by various health technology assessment bodies [37].

In our data, the PROMIS PF20a score had the strongest correlations with the R-PAct scale and 6MWD, both in cross-sectional and longitudinal measurements. This correlation between PROMIS PF20a and R-PAct scores was expected, as the questionnaires evaluate physical performance in similar domains, such as taking a shower and doing yard work [13, 22]. PROMIS PF20a scores had the weakest correlation with % predicted FVC, but nevertheless, moderate correlations were observed, and a statistically significant association was observed between the 2 outcomes when combining all longitudinal measurements in a multivariable, mixed-effects, linear regression model.

To improve our understanding of how changes in PROMIS PF20a scores should be interpreted in clinical practice, the MCID of PROMIS PF20a was estimated using both a distribution-based and an anchor-based approach, the anchor being the SGIC overall physical well-being score. This anchor was selected as it directly measures clinical improvement in physical well-being in patients and had a significant albeit weak correlation with the PROMIS PF20a score. Depending on the chosen method, derived MCIDs ranged between 2.2 and 4.2 in our study, which is in line with the MCID reported in a systematic review and meta-analysis of PROMIS measures in other populations [38].

Various checklists have been developed to operationalize tools that measure PROs, such as the validation checklist proposed by Francis et al. [19]. In the analyses presented in this manuscript, “construct validity” and “scoring and interpretation” were evaluated in LOPD (Table 4), which are considered key domains of PRO validity. Our data support expected associations with existing PRO measures and other relevant outcomes at single time points. In addition, the questionnaire can measure changes over time (construct validity). Providing the MCID will add to the interpretation of PROMIS PF20a scores and will facilitate to measure individual disease progression and/or therapy benefit in patients with LOPD.

**Table 4:** Validation checklist for the PROMIS physical function questionnaire

Validation category	Validation element
Conceptual model	• Has the PRO construct to be measured been specifically defined?
	• Has the intended respondent population been described?
	• Does the conceptual model address whether a single construct/scale or multiple subscales are expected?
Content validity	• Is there evidence that members of the intended respondent population were involved in the PRO measure’s development?
	• Is there evidence that content experts were involved in the PRO measure’s development?
	• Is there a description of the methodology by which items/questions were determined (e.g., focus groups, interviews)?
Reliability	• Is there evidence that the PRO measure’s reliability was tested (e.g., test-retest, internal consistency)?
	• Are reported indices of reliability adequate (e.g., ideal: *r* ≥ 0.80; adequate: *r* ≥ 0.70; or otherwise justified)?
Construct validity	• Is there reported quantitative justification that single scale or multiple subscales exist in the PRO measure (e.g., factor analysis, item response theory)?
	• **Are there findings supporting expected associations with existing PRO measures or with other relevant data?**
	• Are there findings supporting expected differences in scores between relevant known groups?
	• **Is the PRO measure intended to measure change over time? If yes, is there evidence of both test-retest reliability and responsiveness to change?**
Scoring and interpretation	• Is there documentation how to score the PRO measure (e.g., scoring method such as summing or an algorithm)?
	• Has a plan for managing and/or interpreting missing responses been described (i.e., how to score incomplete surveys)?
	• **Is information provided about how to interpret the PRO measures scores [e.g., scaling/anchors, (what high and low scores represent), normative data, and/or definition of severity (mild or severe)]?**
Respondent burden and presentation	• Is the time to complete reported and reasonable? Or, if it is not reported, is the number of questions appropriate for the intended application?
	• Is there a description of the literacy level of the PRO measure?
	• Is the entire PRO measure available for public viewing (e.g., published with citation, or information provided about how to access a copy)?

The validation checklist was retrieved from Francis et al. [19], Fig. 1. Bold items were evaluated in the analyses presented in this manuscript. The original checklist published in Systematic Reviews [19] was distributed under terms of the Creative Commons Attribution 4.0 International License (http://creativecommons.org/licenses/by/4.0/); no changes were made

PRO: patient-reported outcome

Other characteristics on the validation checklist have been answered previously by PROMIS or other studies. The conceptual model has been described well: PROMIS PF20a measures physical function in an intended respondent population of patients with LOPD, using 2 subscales, both ranging from 0 to 5 (5 = “without any difficulty” or “not at all” to 1 = “unable to do” or “cannot do”) [22].

PROMIS questionnaires have been developed and validated by experts using rigorous methodology; the used methodology, including the use of literature searches, focus groups, and interviews, is described on the PROMIS website [39]. Previous studies have successfully tested the reliability of PROMIS physical function measures in cancer patients [40].

Construct and content validity, and reliability of PROMIS questionnaires, including physical function, has been evaluated previously in a small-scale (*N* = 30), cross-sectional study of patients with LOPD [21]. Our data now analyzed multiple PROMIS PF20a assessments in a larger cohort (*N* = 122) of patients with LOPD, allowing longitudinal evaluation of this PRO measurement questionnaire, and therefore, further validating the use of PROMIS PF20a in patients with LOPD. Our data support expected associations between PROMIS PF20a and other functional measures in the patient population. Expected differences in PROMIS PF20a between relevant known groups are corroborated by data from the PROPEL study showing that patients in the 2 treatment arms had differences in PROMIS PF20a scores (with a least square mean difference [95% confidence interval] of 1.9 [-1.5, 5.3]), which was similar to differences in other outcome measurements of physical functioning [6].

Finally, the domains scoring and interpretation, and respondent burden and presentation have been positively evaluated too. The questionnaire is available for public viewing [22] and has been used in patients with a high level of education and in patients with an education level below high school [40, 41]. PROMIS has created documentation explaining how to score the questionnaire and how to handle missing values. PROMIS works actively with communities to have its questionnaire translated in other languages than English, such as Spanish [41, 42]. Since the short form only contains 20 questions with 5 answering options, the time to complete this questionnaire is reasonable and comparable to R-PAct (18 questions with 3 answering options) [13], PDSS (12 questions that are answered on a scale from 0 [none] to 10 [as bad as I can imagine]), and PDIS (15 questions on mobility-related physical activities and mood in the past 24 h, with scales varying from 0 to 3 to 0 to 10) [20, 43].

Combining these findings, we consider that PROMIS PF20a has validity as a PRO measurement tool in patients with LOPD. Future studies are needed to compare PROMIS PF20a to other PRO measurement tools such as R-PAct, PDIS, and PDSS. A strength of PROMIS PF20a compared to these other tools is that PROMIS PF20a is already used in other diseases, and that the score can be computed to a T-score metric, where 50 represents the average score for the general population in the United States and 10 is one standard deviation [44]. This allows comparison of the physical function of a patient with LOPD with the average general population and patients with other diseases.

### Limitations

The PROPEL study was not designed to validate PROMIS PF20a, and therefore, the analyses presented in this manuscript should be regarded as secondary, exploratory analyses. However, the advantage of using PROPEL data was that we were able to evaluate PROMIS PF20a in a reasonably-sized cohort, considering the rarity of LOPD. Another limitation of the analyses is that follow-up scores of PRO measurement tools such as PROMIS PF20a are inevitably associated with baseline scores: a greater level of disability at baseline provides more opportunity for improvement than for deterioration. In our analysis, this seems quite balanced as the MCID for improvement (2.4) was relatively similar to the MCID for worsening (3.4).

Potential limitations of the use of MCIDs have been described in detail by Copay et al. [45]. A limitation of using SGIC overall well-being as an anchor is that recall bias may have occurred when patients recalled their health state of a year ago [46, 47]. SGIC is, nevertheless, considered a useful anchor in general as it directly asks patients whether they had observed a meaningful improvement (or worsening) as compared to baseline; in our study, it is important to note that SGIC is significantly, but only weakly correlated with PROMIS PF20a change from baseline at week 52. Moreover, the distribution-based MCIDs in general are not theoretically or empirically tied to any patient-reported experience. Hence MCIDs– whether anchor or distribution-based– in this study are subject to a high degree of uncertainty and should be interpreted cautiously.

Psychometric evaluation might be needed to further support the validation of PROMIS PF20a. Furthermore, considering that LOPD is a slowly progressing disease, longer term evaluation may provide more information on the use of PROMIS PF20a to assess changes in physical function over time.

## Conclusions

In patients with LOPD, PROMIS PF20a scores generally correlated well to the 6MWD, % predicted FVC, MMT of lower extremities, GSGC, and R-PAct scores when measured at various time points, and to a lesser extent when evaluating changes in functioning over time. The MCID ranged between 2.2 and 4.2 using different methodological approaches. These results and previously published data on PROMIS PF20a indicate that the questionnaire has validity and is a valuable, easy-to-use and readily available tool to measure physical function in patients with LOPD. Its use should be considered in future clinical studies of patients with LOPD and potentially in clinical practice.

### Electronic supplementary material

Below is the link to the electronic supplementary material.

**Supplementary Material 1:** Supplementary data

**Supplementary Material 2:** Plain language summary

## Data Availability

Data sharing proposals and requests for data of the PROPEL study will be reviewed on a case-by-case basis. Requests for data should be addressed to Mitchell Goldman at mgoldman@amicusrx.com. Requests will be reviewed by a medical steering committee.

## Abbreviations

6MWD: 6-minute walk distance
BMI: Body mass index
COSMIN: Consensus-based Standards for the selection of health Measurement Instruments
ERT: Enzyme replacement therapy
FVC: Forced vital capacity
GAA: Acid α-glucosidase
GSGC: Gait, stairs, Gowers’ maneuver, chair
ISOQOL: International Society for Quality-of-Life Research
LOPD: Late-onset Pompe disease
MCID: Minimal clinically important difference
MMT: Manual muscle test
NHANES: National Health and Nutrition Examination Survey
PDIS: Pompe Disease Impact Scale
PDSS: Pompe Disease Symptom Scale
PF20a: Physical Function short form 20a
PRO: Patient-reported outcome
PROMIS: Patient-Reported Outcome Measurement Information System
rhGAA: Recombinant human acid α-glucosidase
R-PAct: Rasch-built Pompe-specific Activity scale
SD: Standard deviation
SF-36: Medical Outcomes Study 36-Item Short-Form Health Survey
