# Supplementary material for: Validation of the Patient-Reported Outcomes Measurement Information System (PROMIS®) physical function questionnaire in late-onset Pompe disease using PROPEL phase 3 data
Source: J Patient Rep Outcomes. 2024 Jan 31;8:13. doi: 10.1186/s41687-024-00686-z (PMC10830974; doi:10.1186/s41687-024-00686-z)
Supplement: Supplementary file 1 — Supplementary Material 1: Supplementary data [file 41687_2024_686_MOESM1_ESM.docx]

# Supplementary Data

### Supplementary table 1. Summary of PROPEL baseline data.

|  | **Cipaglucosidase alfa plus miglustat** | **Alglucosidase alfa plus placebo** | **Total** |
| --- | --- | --- | --- |
| Number of patients | 85 | 38 | 123 |
| Age |  |  |  |
| mean, SD | 48 (13) | 45 (13) | 47 (13) |
| median, range | 48 (19-74) | 46 (22-66) | 47 (19-74) |
| Sex, female (%) | 49 (58%) | 18 (47%) | 67 (55%) |
| Race |  |  |  |
| White | 74 (87%) | 30 (79%) | 104 (85%) |
| Asian | 5 (6%) | 5 (13%) | 10 (8%) |
| Other | 6 (7%) | 3 (8%) | 9 (7%) |
| Previous ERT status, ERT naïve (%) | 20 (24%) | 8 (21%) | 28 (23%) |
| Body mass index |  |  |  |
| mean, SD | 25 (5) | 27 (7) | 25 (6) |
| median, range | 24 (13-36) | 24 (16-47) | 24 (13-47) |

ERT: enzyme replacement therapy; SD: standard deviation.

### Supplementary figure 1. Correlation between PROMIS physical function short form 20a scores and other outcome measures, unadjusted model.


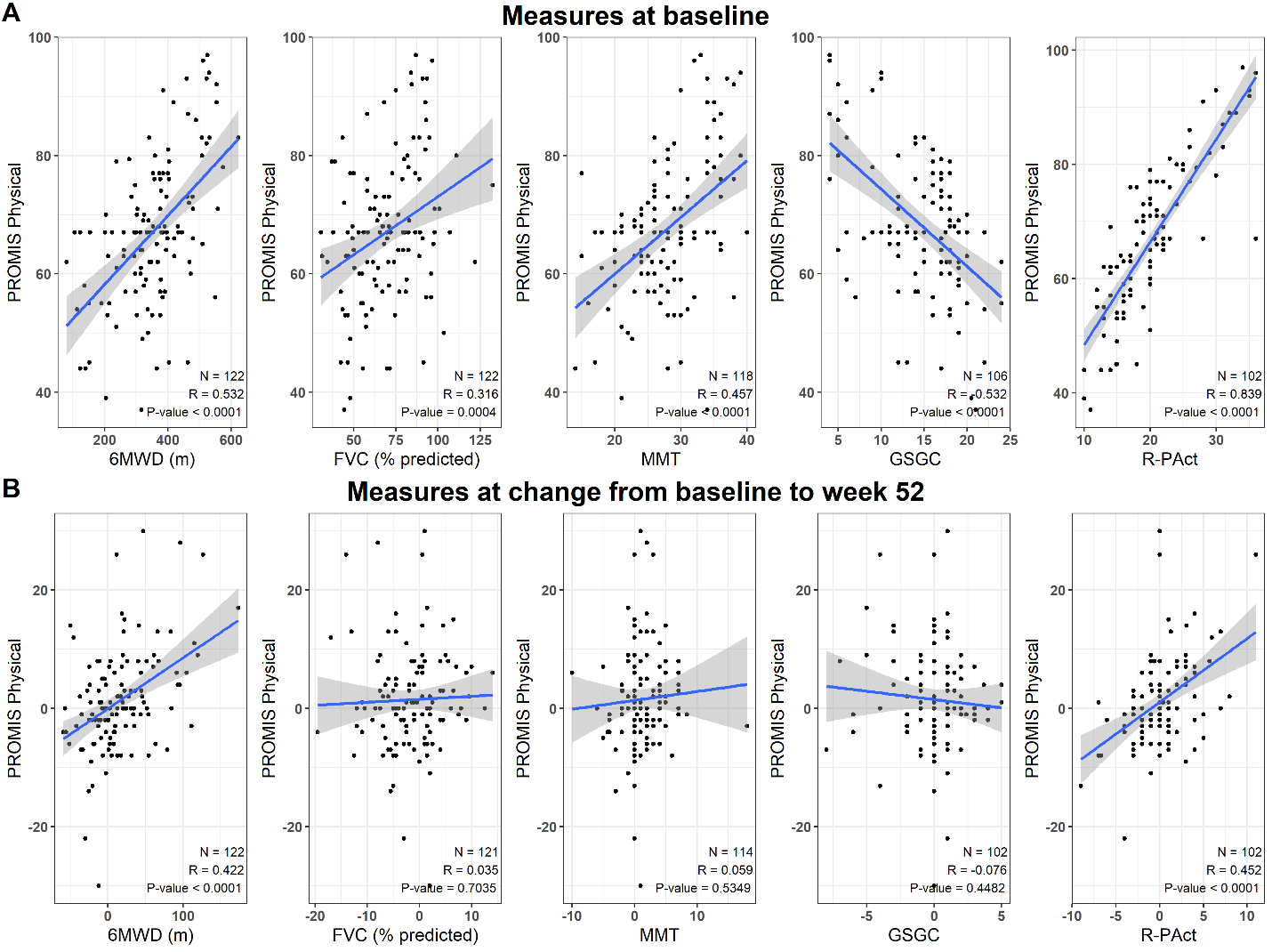


6MWD: six-minute walk distance; FVC: forced vital capacity; GSGC: gait, stairs, Gower’s maneuver, chair; MMT: manual muscle test; N: number of patients; R: Pearson correlation coefficient; R-PAct: Rasch-built Pompe-specific Activity scale.
