# Supplementary material for: Validation of the Patient-Reported Outcomes Measurement Information System (PROMIS®) physical function questionnaire in late-onset Pompe disease using PROPEL phase 3 data
Source: J Patient Rep Outcomes. 2024 Jan 31;8:13. doi: 10.1186/s41687-024-00686-z (PMC10830974; doi:10.1186/s41687-024-00686-z)
Supplement: Supplementary file 2 — Supplementary Material 2: Plain language summary [file 41687_2024_686_MOESM2_ESM.docx]

# Plain English summary

Late-onset Pompe disease (LOPD) is a rare, hereditary disease. Patients with LOPD have decreased production of an enzyme, which leads to symptoms that gradually get worse, including muscle weakness and trouble breathing. Enzyme-replacement therapy may slow down the disease progression. In recent years, enzyme-replacement therapies have been improved. To measure the benefit of such new therapies, patients with LOPD are asked to fill in surveys about their symptoms before and during treatment, but there is no standard survey to use. In this study, we studied a survey called the Patient-Reported Outcome Measurement Information System (PROMIS) Physical Function short form 20a (PF20a) questionnaire. This questionnaire is used for various diseases and tests someone’s ability to perform daily physical activities such as getting dressed. We compared the results of this survey to other tests which evaluate a variety of functions, such as how far a patient can walk in 6 minutes, leg muscle strength, and a patient’s lung capacity. In general, we found that the score provided by the PROMIS PF20a questionnaire had moderate to strong agreement with other test scores. Furthermore, we looked at the minimum difference in PROMIS PF20a scores that a patient found a relevant difference (i.e., a relevant improvement or worsening of the disease). Patients found a difference between 2.4 and 4.2 points in the score relevant. The results from this study show that PROMIS PF20a may be used to measure symptoms and follow symptoms over time in patients with LOPD.
